# Osseointegration Following Transfemoral Amputation After Infected Total Knee Replacement: A Case Series of 10 Patients With a Mean Follow-up of 5 Years

**DOI:** 10.1016/j.artd.2022.04.008

**Published:** 2022-05-21

**Authors:** Muhammad Adeel Akhtar, Jason Shih Hoellwarth, Kevin Tetsworth, Atiya Oomatia, Munjed Al Muderis

**Affiliations:** a Trauma and Orthopaedic Department, Victoria Hospital Kirkcaldy, NHS Fife, Kirkcaldy, Scotland, UK; b Limb Salvage and Amputation Reconstruction Center, Hospital for Special Surgery, New York, NY, USA; c Department of Orthopaedic Surgery, Royal Brisbane and Women’s Hospital, Queensland, Australia; d Limb Reconstruction Centre, Macquarie University Hospital, Macquarie University, Macquarie Park, Australia

**Keywords:** osseointegration, amputation, total knee arthroplasty, total knee infection, above knee amputation, knee arthrodesis

## Abstract

**Background:**

Management of total knee replacement (TKR) infection may sometimes prompt knee fusion (KF) or transfemoral amputation (TFA), both associated with low mobility and quality of life (QOL). Transcutaneous osseointegration for amputees provides superior mobility and QOL vs traditional socket prostheses but has not been studied for patients with a history of infected TKR. This study investigates the following hypothesis: Patients who have had TFA or KF following infected TKR achieve better mobility and QOL following transfemoral osseointegration.

**Material and methods:**

A retrospective evaluation of the prospectively maintained registry identified 10 patients who had prior infected TKR. The mobility assessments (patient daily prosthesis wear time, K-level, Timed Up and Go, 6-Minute Walk Test) and QOL surveys (Questionnaire for Persons with a Transfemoral Amputation Global, Mobility, and Problem scores) were compared preoperatively and after at least 2 years. Complications requiring an additional surgery were also evaluated.

**Results:**

Daily wear hours, K-level, and 6-Minute Walk Test and Questionnaire for Persons with a Transfemoral Amputation Global and Problem scores significantly improved (*P* < .05). Through 1 year, 4 patients (40%) had additional surgeries. After several years, 7 patients (70%) had at least 1 additional surgery, and 5 (50%) had multiple, for an average of 1 debridement and 1.3 soft-tissue refashionings per patient. One patient died of newly diagnosed cancer 1 year after transcutaneous osseointegration for amputees.

**Conclusion:**

Transfemoral osseointegration confers significantly better mobility and QOL vs KF or a TFA with traditional socket prostheses following infected TKR. Technique improvements to prevent subsequent surgeries may provide an increasingly streamlined experience.

## Introduction

A serious complication of total knee replacement (TKR) is prosthetic joint infection (PJI). Infected TKRs can be salvaged with surgical debridement and partial or total component revision, but sometimes they may require full component removal with knee fusion (KF) or transfemoral amputation (TFA) [[1], [2], [3]]. The prognosis following TKR PJI is dismal: 21% of all TKR PJI patients die within 5 years even if they retain their leg [4]. For patients requiring TFA, the average lifespan is 2.6 years [5]; only 25% of the amputees are able to walk with a traditional socket prosthesis (TSP) [6], and fewer than 10% consider themselves functionally independent [5]. The TSP is a major factor for low mobility and quality of life (QOL) for these patients because of the myriad of challenges such as skin breakdown and fit problems [[7], [8], [9], [10]].

**Figure 1:**
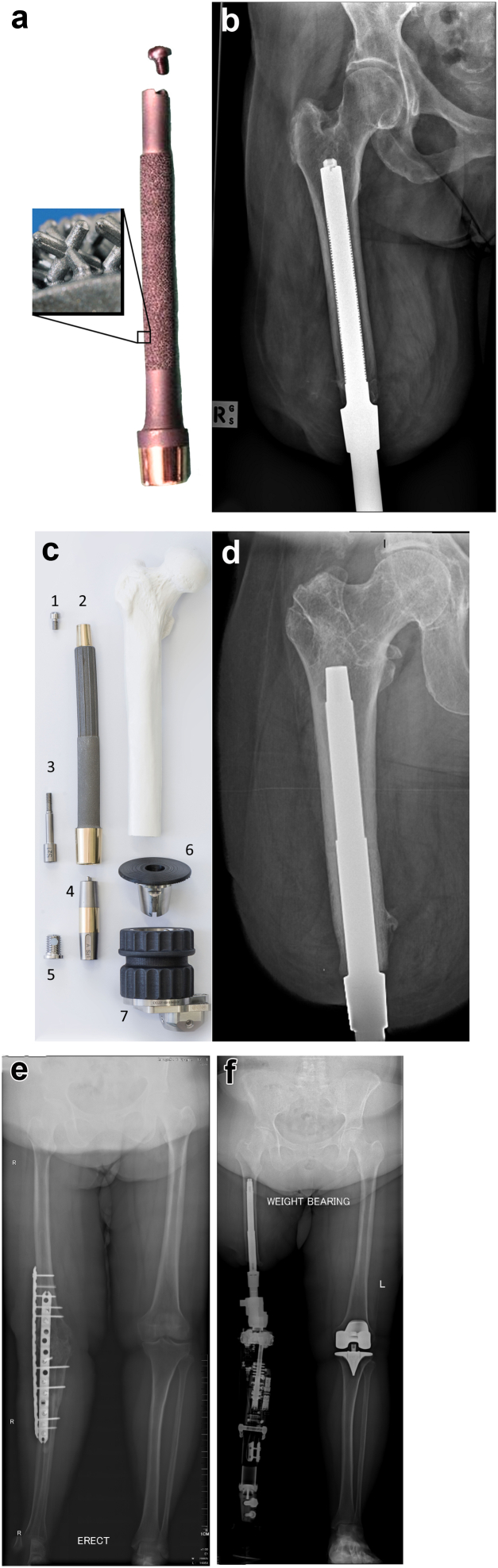
(a) ILP implant showing a zoomed in box of the surface texture. (The main implant photograph is adapted with permission from Springer Nature: Springer Nature, Operative Orthopädie und Traumatologie. Aschoff HH, Clausen A, Tsoumpris K, Hoffmeister T. Implantation der Endo-Exo-Femurprothese zur Verbesserung der Mobilität amputierter Patienten. Oper Orthop Traumatol. 2011 Dec;23 [5]:462-72. The zoom-in box of ILP texture is adapted, with permission, from Springer Nature: Springer Nature, Der Orthopäde. Juhnke DL, Aschoff HH. Endo-Exo-Prothesen nach Gliedmaßenamputation. Der Orthopäde. 2015 Jun; 44 [6]:419-25. Epub 2015 May 14). (b) Anterior-posterior right femur radiograph showing the ILP. (c) Exploded view of the OPL system, with the components arranged at approximately the proximal-distal levels in which they would be once assembled and implanted in a transfemoral amputee. 1, Proximal cap screw; 2, OPL body; 3, safety screw; 4, dual cone abutment adapter; 5, permanent locking propeller screw; 6, proximal connector; and 7, prosthetic connector. (d) Anterior-posterior right femur radiograph of the OPL. (e) Long-standing radiograph of patient 2 with a fused knee before and (f) after TFA with TOFA. Both the ILP and OPL have an intramedullary body textured to facilitate bone interdigitation, a flat abutment, a smooth transcutaneous collar to prevent skin adhesion, and the collar mates with the dual cone which connects an external prosthetic limb. Major ILP vs OPL differences are the metal used (cobalt-chrome alloy vs Ti6Al4V titanium alloy); the ILP is not textured to promote both interdigitation at the 1.5-cm portion near the abutment, whereas the OPL texturing includes the flat abutment; the texture shape and depth (1.5-mm uniform Czech hedgehog vs 0.5-mm variable bump texture); and the OPL’s proximal taper can mate with a specified arthroplasty attachment; Video 1. This video of patient 9 shows a typical gait of a transfemoral amputee using a socket prosthesis. The patient must dip her right hemipelvis to sink into the prosthesis to improve stance stability and then must elevate her right hemipelvis to lift the prosthesis which sags due to the skin motion. Her torso has substantial side-to-side sway during this process. Despite her being diligent with her rehabilitation, she still shows evidence of poor balance, moving her arms in response to moments of unsteadiness. One year following TOFA, the same patient demonstrates a markedly improved gait. She does not need to elevate or dip her right hemipelvis because the prosthesis is not loose. She therefore does not need to sway her trunk left and right. She is comfortable turning on the affected side. Her balance is excellent, as she does not need to use her arms to regain balance. ILP, integral limb prosthesis; OPL, osseointegrated prosthetic limb.

Transcutaneous osseointegration for amputees (TOFA) (Fig. 1) has revolutionized amputee rehabilitation by obviating socket-interface problems [11]. TOFA has been performed predominantly for transfemoral amputees following traumatic injuries [12,13] and consistently provides superior prosthetic daily wear hours, proximal joint range of motion, K-level, and QOL compared to TSP rehabilitation, while reducing ambulation energy consumption [[14], [15], [16], [17]]. Video 1 shows the gait difference for a patient using a TSP and following TOFA. Historically, TOFA was assumed to be too risky for some patient populations although several recent studies have identified that press-fit TOFA is in fact beneficial for many of such populations [[18], [19], [20]]. Patients with a history of TKR PJI resulting in TFA or KF is another population that has been generally overlooked and not previously studied.

To address this knowledge gap, we evaluated a cohort of 10 patients to investigate the following hypothesis: Patients who have had TFA or KF following infected TKR achieve better mobility and QOL following TOFA. We also evaluated associated complications. All patients were followed up for at least 2 years or until they died.

## Material and methods

The preoperative assessment, operative technique, postoperative follow-up routines, rehabilitation principles, and implant principles have been previously detailed [11,21,22] and summarized in the following sections.

### Study design

After institutional ethics review, we retrospectively reviewed our prospectively maintained TOFA database. A manual review was performed of all initial consultation notes to include those who had a history of TKR and had either been followed up for at least 2 years following TOFA or died prior to 2 years.

#### Participants

In general, and also for the patients evaluated in this study, patients considered for TOFA are skeletally mature adults who (1) report pain or mobility dissatisfaction with their TSP; (2) have an intact limb with incapacitating pain, deformity causing functional impairment, or profound distal weakness, whose functional capacity is considered likely to be improved by amputation; or (3) are recent amputees preferring TOFA to TSP rehabilitation. Specific inclusion criteria for this study were patients with a history of ipsilateral TKR prior to the TOFA and a follow-up period of at least 2 years (or having died prior to 2 years); coincidentally, all such patients had infection as the fundamental TKR complication (as opposed to other causes such as periprosthetic fracture, aseptic loosening, or instability). Patients without prior ipsilateral TKR were excluded. These criteria yielded 10 patients (summarized in Table 1). Chart review focused on demographic information, preoperative and postoperative mobility and QOL survey outcomes, and post-TOFA complications. The TFA could have been performed synchronously (for patients with KF) or metachronously (for patients with prior TFA) to the TOFA.

**Table 1:** Patient presentation summary.

Pt #	Age/sex/side	BMI	Knee surgical history in relation to pTKR^a^	Presenting status, residual femur cm (if amputee), years since surgery, current mobility	Follow-up years
1	48/Female/left	39.2	Meniscectomy (−18), high tibia osteotomy (−7), **pTKR**, rTKR (+1), rTKR (+1.5), TFA (+2).	Amputee, 26 cm, 15 y, wheelchair	9.7
2	60/Female/right	39.1	**pTKR**, rTKR (+1.5), scope (+1.75), patellectomy (+2), rTKR (+2.5), KF (+3).	Fused knee, 2 y, wheelchair	5.6
3	55/Male/right	37.6	ACL (−2), **pTKR**, rTKR (+1), rTKR (+2), rTKR (+3), TFA (+4)	Amputee, 31 cm, 3 y, wheelchair	7.4
4	65/Male/left	30.0	**pTKR**, spacer (+1), rTKR (+3), TFA (+5)	Amputee, 20 cm, 8 y, wheelchair	6.6
5	75/Male/right	36.8	Unknown knee surgery (−27), scope (−9), **pTKR**, explant and multiple debridements (+11), KF with (+12), TFA (+13)	Amputee, 18 cm, 6 y, socket	5.1
6	71/Male/right	30.0	**pTKR**, scope (+0.5), rTKR (+1.5), rTKR (+2.5), KF (+3.5), TFA (+5.5)	Amputee, 28 cm, 6 y, wheelchair	5.6
7	53/Female/right	38.7	UKR (−4), muscular flap coverage (−0.5), **pTKR**, rTKR (+0.5), polyexchange (+0.5), KF (+3), TFA (+5)	Amputee, 25 cm, 2 y, socket	5.2
8	78/Male/left	26.1	**pTKR**, rTKR (+2), KF (+2.5)	Fused knee, 4 y, front walker	3.6
9	46/Female/right	39.9	**pTKR**, rTKA +6 debridements +4 spacers (+0.5-1), TFA (+1.5)	Amputee, 22 cm, 1 y, socket	2.5
10	73/Male/left	19.9	THR (−10), THR removal for fracture (−3), **pTKR**, KF (+1)	Fused knee, 4 y, wheelchair	1
All	62.4 ± 11.4 6/10 male	33.7 ± 6.9	n/a	7 Amputees, 3 fused, 5.1 ± 4.1 y since last surgery, 6 wheelchair, 4 socket	5.2 ± 2.5

BMI, body mass index; KF, knee fusion; pTKR, primary total knee replacement; rTKR, revision total knee replacement; scope, arthroscopy; spacer, explantation with antibiotic spacer; TFA, transfemoral amputation; THR, total hip replacement UKR, unicompartmental knee replacement; ACL, anterior cruciate ligament reconstruction.

All patients experienced a total knee infection prompting the subsequent interventions. All procedures are named (followed by the timing, in years, before or after the pTKR).

a This column presents the patient’s relevant surgical knee history. All known procedures are listed.

All TOFA patients, including those in this study, have a thorough physical examination to identify potential issues: in this population, evidence of persistent infection such as sinus tracts or cellulitis. Radiographic examination includes radiographs to clarify anatomy and evidence of persistent infection such as osteomyelitis. All also have preoperative laboratory screening that includes infectious markers (erythrocyte sedimentation rate, C-reactive protein, white blood cell count with differential); patients with abnormal values have source evaluation and control prior to TOFA. None of this cohort’s patients had any such evidence of persistent infection.

### Study outcomes

#### Functional outcomes

At the first consultation, patients interested in TOFA were asked to complete QOL surveys (Questionnaire for Persons with a Transfemoral Amputation [QTFA] [23] and other custom questions) and perform basic mobility tests (Timed Up and Go [TUG] [24] and 6-Minute Walk Test [6MWT] [25]); their K-level [26] was determined by the surgeons during examination. The patients were asked to complete the same questionnaires and mobility tests at annual follow-up visits where their K-level was reassessed by the surgeon. Surgery and postoperative care were not withheld from patients who declined to complete the mobility tests and surveys. Patients were encouraged to remain local for at least 3 months postoperatively for focused rehabilitation.

#### Adverse events

Adverse events related to the patient’s status as an osseointegrated amputee were recorded, specifically any additional surgery to the ipsilateral extremity. These included infection (debridement with implant retention or removal), periprosthetic fracture, and refashioning (a soft-tissue debulking procedure akin to tissue tightening or redraping, removing skin and excess fat aimed at stabilizing the tissue overlying the muscles that form the transcutaneous stoma). Systemic events related to the surgery and status as an osseointegrated amputee (such as cardiac or pulmonary events) were investigated; none occurred.

### Data analysis

Statistical calculations were performed using Google Sheets (Google LLC, Mountain View, CA) and the XLMiner Analysis ToolPak (Frontline Systems, Incline Village, NV). Frequencies were compared using Fisher’s exact test. Means were compared using Student’s t-test. Differences were considered significant at *P* < .05.

The following data irregularities occurred. Patient 5 declined to participate in the mobility tests. Patient 9 did not report preoperative prosthesis wear hours. Patient 10 died 12 months after TOFA due to newly diagnosed pancreatic cancer, precluding the collection of follow-up data. We retained these patients in this study for 2 reasons. First, because there are extremely limited published data on this topic, so we feel each treated patient offers meaningful insight, particularly because complications were tracked. Second, we feel this better represents the true clinical expectations because we do not refuse TOFA to patients who decline to fulfill all research-data-collection requests.

## Description of TOFA surgery and rehabilitation

### Press-fit implants

Our practice used an integral limb prosthesis (Orthodynamics, Lubeck, Germany) early on and has transitioned to the osseointegrated prosthetic limb (Permedica Medical Manufacturing, Lecco, Italy). The TOFA implant systems are specifically designed for press-fit fixation and consist of 2 components placed at surgery: an intramedullary nail-type piece that achieves skeletal integration, and a dual cone adapter that inserts into the intramedullary component to interface with the external prosthetic limb. Both systems are shown and described in the legend of Figure 1. Previous publications describe these implants in great detail [11]. Both systems achieve immediate stability to allow progressive weight-bearing as bone remodeling progresses.

### Surgical procedure

**Figure 2:**
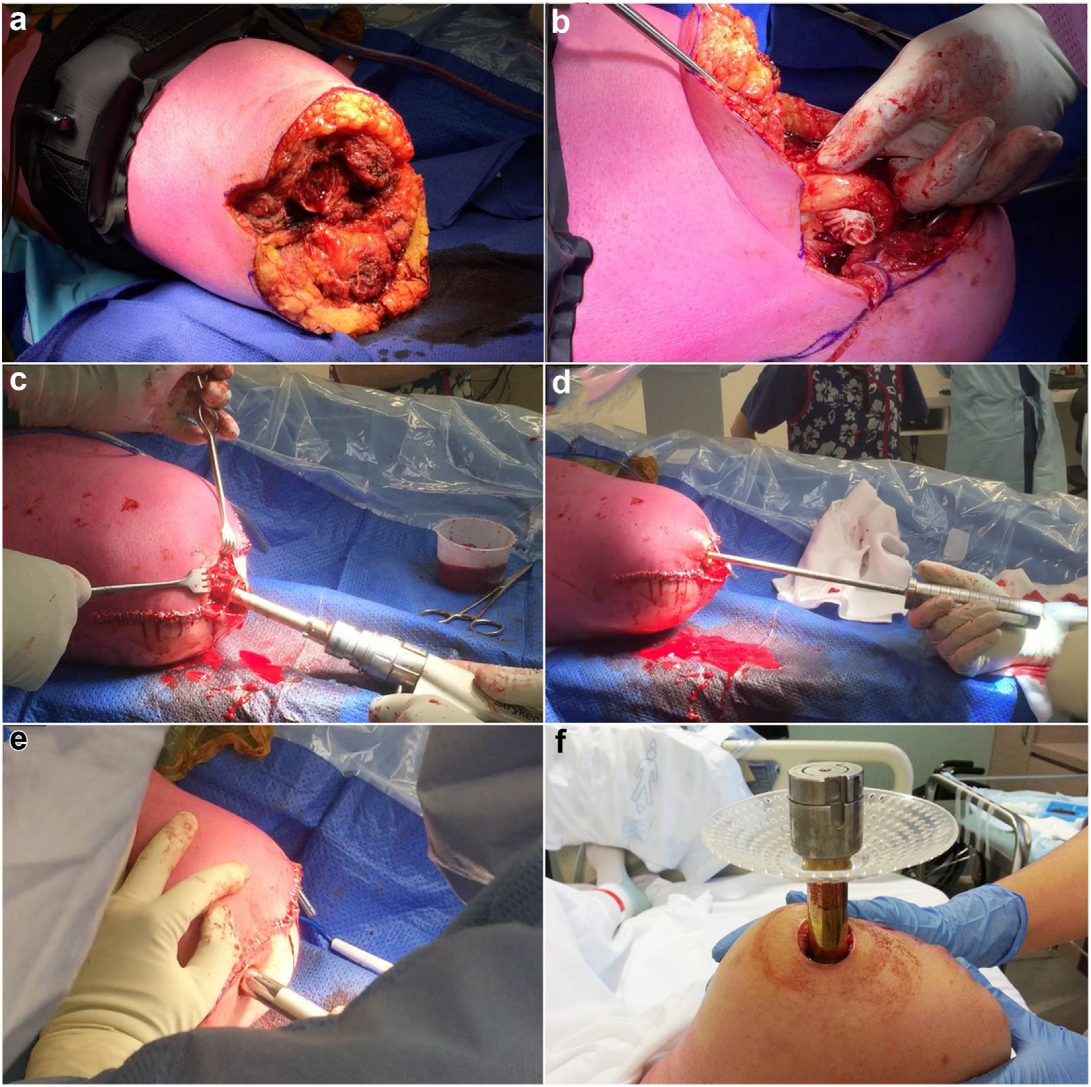
Key surgical steps for single-stage TOFA per the Osseointegration Group of Australia Accelerated Protocol-2. (a) Exposure. A percutaneous exposure can be utilized in situations where there is minimal, well-contoured soft tissue with easy access to the residual bone and when nerve procedures are not indicated. For most patients, a wide exposure is necessary. A wide exposure allows excision of heterotopic ossification, removal of redundant fat and muscle (refashioning), and other potentially necessary adjunctive procedures. (b) One common category of adjunctive procedures is to address nerve pain or symptomatic neuromas, either prophylactically or therapeutically. This panel shows the identification of a large symptomatic neuroma. Targeted muscle reinnervation or regenerative peripheral nerve interface can be performed during the TOFA surgery. (c) Once the residual bone and soft tissue are prepared, intramedullary reaming can be performed. This process is fundamentally similar to reaming for an intramedullary nail. However, unlike a fracture nail, one must be careful to not overream. Since a press-fit implant relies on extensive intimate contact to achieve immediate stability, the surgeon should be attentive to the bone chatter. Achieve the closest center-center placement of the guidewire. A uniform long corridor of bone is ideal: Gently advance the reamer, withdrawing often to clear flutes to avoid burning the bone. Try to minimize “jumps” or hard wobbles in the reamer, by advancing and upsizing slowly. An excellent corridor is often achieved once the reamer has a stable and uniform sound and feel as it advances through much of the path: no skips, jumps, or wobbles. As the reamer approaches the templated diameter of the available implants, the broach can be used to make smaller progressions to fit an implant with much less risk of excessive widening due to overreaming. (d) Upon achieving a uniform corridor, the implant can be inserted. This frame shows the implant is tucked flush with the skin, held by the handle impactor. Like a press-fit joint replacement implant, there should be some reasonable resistance to seat the implant. Some experience is necessary to learn the appropriate amount of resistance. An oversized implant can cause an extensive fracture. It is common to have small nonpropagating fractures at the distal centimeter of the bone, particularly in long-term amputees with osteoporotic bone. They have proven clinically inconsequential and should not be fixed with cerclage, plate, or other devices. So long as the fractured fragment remains alive (in contact with other bone or muscle), they achieve stability on the TOFA implant. In our experience, propagating fractures have not occurred. The implant should be impacted until the flat abutment contacts the prepared, flat distal end of the bone. (e) A transcutaneous “stoma” is necessary to pass the dual cone external prosthesis connector. This panel shows a different patient from the prior panels. In the prior panels, the incision was placed so that the unclosed portion became the stoma. In this patient shown in the panel, the incision was pulled over the top for closure, and a percutaneous circular cut was made to accommodate the dual cone. Both techniques are suitable. (f) The immediate postoperative appearance of a stoma. The dual cone penetrates the skin, and the prosthesis adapters are present distally. A plastic disk “tulip” can be placed to hold gauze to absorb the mild serous leakage that can occur for some patients until the stoma matures.

The procedure is performed in a single stage as described in the published protocol [22]. Immediate preincision prophylactic intravenous antibiotics are administered (cefazolin unless allergy mandates otherwise). There are 6 key portions of the procedure, as highlighted in Figure 2. There is no true minimum necessary length although approximately 16 cm from the piriformis fossa is ideal to accommodate a standard-length implant; short femurs can be optimized if necessary [27]. There is no maximum length, but 2 principles guide an optimal length: first, the external prosthetic knee level is equal to the contralateral knee (approximately 15 cm minimum distance), and second, the TOFA implant’s abutment rests against the flat cut of the cortices rather than falling into a wide distal metaphyseal flare. Patients with a full leg can have simultaneous amputation with TOFA and soft-tissue contouring. Existing amputees can have either local “percutaneous” implant insertion with negligible bone and soft-tissue dissection or may require more aggressive revision bone amputation and soft-tissue management.

### Rehabilitation

Patients progressed through the postoperative care and rehabilitation protocol, summarized as (1) progressively increasing static axial-only loading directly on the prosthesis abutment within 3 days after TOFA surgery; (2) after half body weight loading has been achieved (around 2-3 weeks), increase axial-only loading using a temporary light-weight prosthesis; and (3) full-weight axial loading with a personalized prosthesis at 4-6 weeks postoperatively. No casts or splints were used. Surgeon follow-up evaluations were scheduled at 3 weeks, 6 weeks, 3 months, 6 months, and annually or as needed.

## Results

### Patient characteristics

Table 1 presents the summarized patient demographic information. Ten patients were evaluated: 6 males and 4 females, 33.7 ± 6.9 years old (range 46-78). All patients had prior TKR PJI. All patients had at least 1 revision TKR prior to their TFA or KF, except patient 10 who directly had KF to manage TKR PJI. All patients had TFA or KF prior to presenting to us. Seven patients presented as amputees (patients 1, 3, 4, 5, 6, 7, 9), 3 of whom had KF prior to amputation (patients 5, 6, 7), and the other 3 presented with a KF (patients 2, 8, 10). Six patients presented wheelchair-bound (patients 1, 2, 3, 4, 6, 10), 3 ambulated using their TSP (patients 5, 7, 9), and patient 8 walked with a front wheeled walker. The time since their last surgery was 5.1 ± 4.1 (range 1-15) years. Eight patients had a body mass index of at least 30. All patients were followed up for at least 2 years, except patient 10 who died at 12 months due to prostate cancer diagnosed after TOFA.

### Functional outcomes

**Figure 3:**
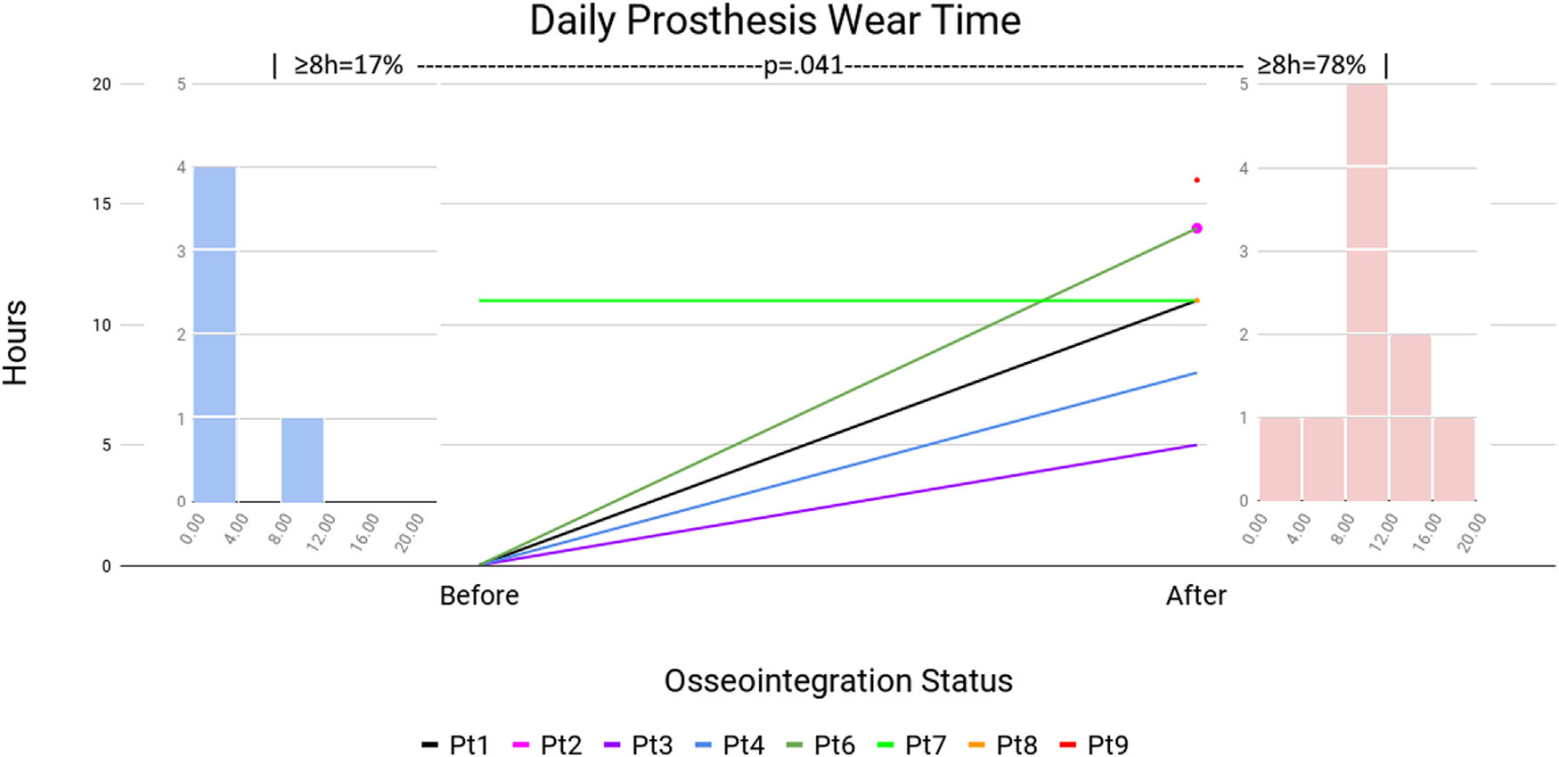
Daily prosthesis wear hours. The center line graph shows each patient as they were in a socket and at their final evaluation following osseointegration. The color identifying each patient is consistent among all figures in this article. The left and right flanking histograms identify the frequency of patient status as they were in a socket and at their final evaluation following osseointegration. It is notable that no patient’s wear hours decreased, and all but 1 patient's wear hours increased. Histograms of patient wear time are presented instead of the mean values because 2 patients were not amputees at initial consultation; a histogram better reflects the true performance change following osseointegration.

**Figure 4:**
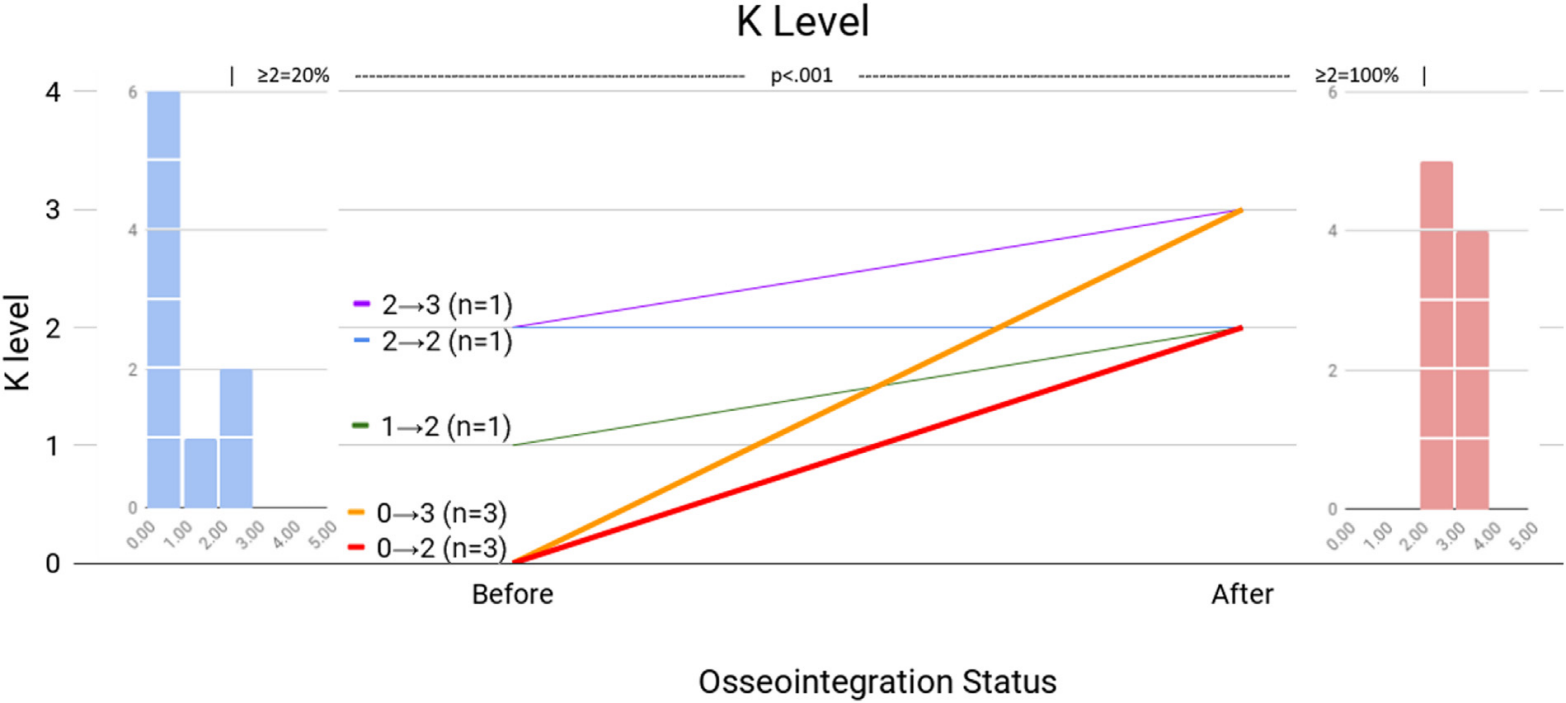
K-level. The center line graph shows each patient as they were in a socket and at their final evaluation following osseointegration. The color identifying each patient is consistent among all figures in this article. The left and right flanking histograms identify the frequency of patient status as they were in a socket and at their final evaluation following osseointegration. It is notable that all patients achieved a K-level of at least 2, including those who were wheelchair-bound at initial consultation.

**Figure 5:**
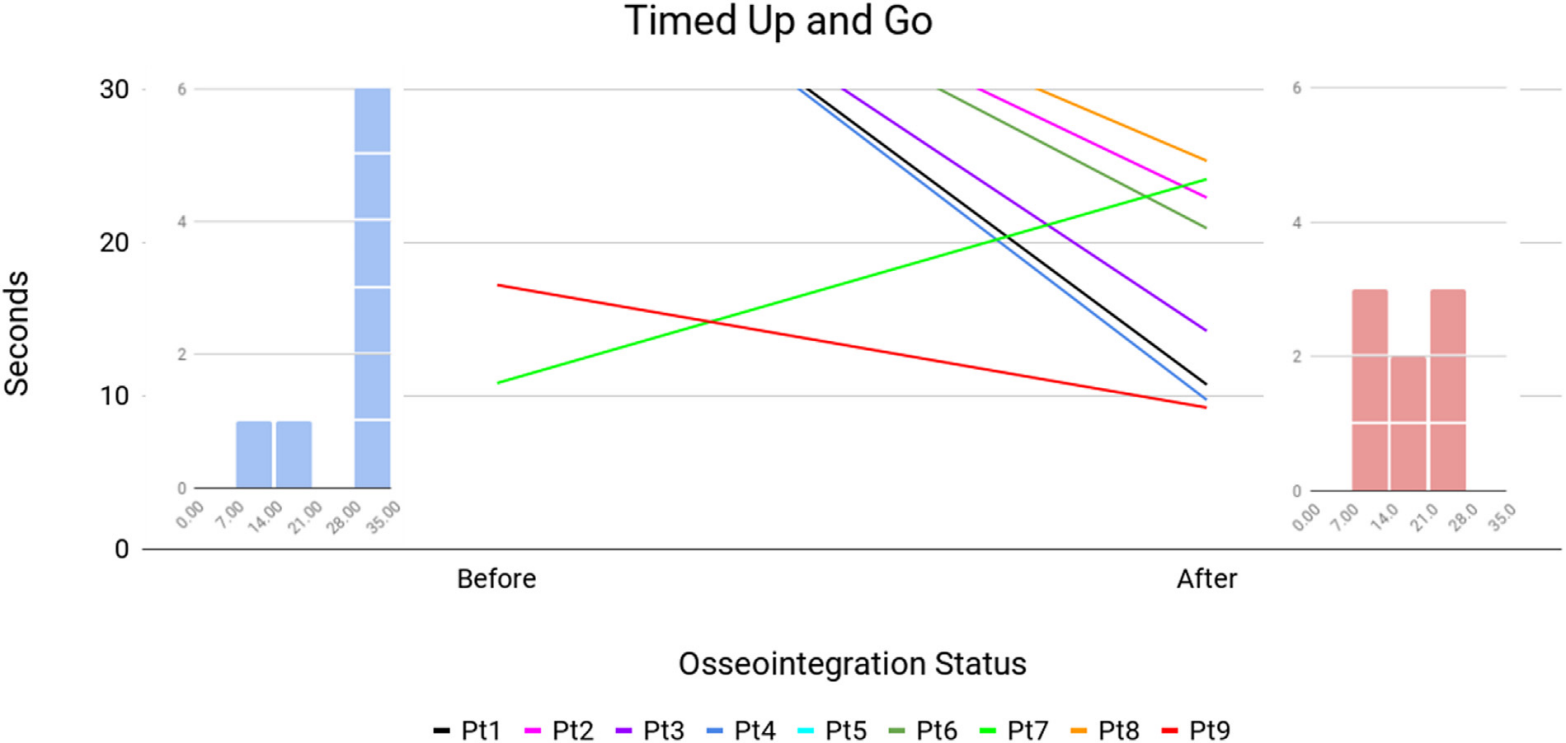
TUG. The center line graph shows each patient as they were in a socket and at their final evaluation following osseointegration. The color identifying each patient is consistent among all figures in this article. The left and right flanking histograms identify the frequency of patient status as they were in a socket and at their final evaluation following osseointegration. To help illustrate the performance improvement of patients who were incapable of performing a TUG prior to osseointegration, they are represented with the lines that start as “off the chart”. It is notable that all patients were able to perform a TUG following osseointegration, including those who were wheelchair-bound prior at their preoperative consultation. Histograms of patient performance are presented instead of the mean values because only 2 patients were able to perform the TUG at initial consultation; a histogram better reflects the true performance change following osseointegration.

**Figure 6:**
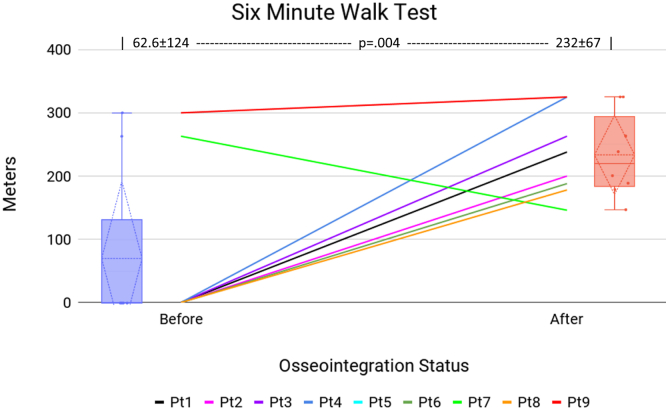
6MWT. The center line graph shows each patient as they were in a socket and at their final evaluation following osseointegration. The color identifying each patient is consistent among all figures in this article. The left and right flanking box plots identify the mean (horizontal dotted line), quartiles (diamond dotted lines), range (whiskers), and each data point (solid dot) of the cohort, representing patient status as they were in a socket and at their final evaluation following osseointegration.

Table 2 presents the patient mobility before TOFA and at the latest follow-up. Patient 5 declined to participate in the mobility tests. Patient 9 did not report preoperative prosthesis wear hours. Patient 10 died prior to any follow-up data collection. The first comparison is prosthesis wear hours (Fig. 3). Three patients (2, 8, and 10) presented with fused knees, not as amputees, so the prosthetic wear hours factor was not applicable. Of the 6 amputees who provided prosthetic wear hours, 1 (17%) wore the prostheses at least 8 hours daily, vs 7 of 9 patients after TOFA (*P* = .041). The next comparison is K-level (Fig. 4). Of the 10 patients, 7 (70%) were wheelchair-bound before TOFA: 4 were amputees (patients 1, 3, 4, 6), and 3 had KF (patients 2, 8, 10). These 7 patients, by definition, were K-level 0 and physically incapable of performing TUG or 6MWT. At the final visit, all 9 living patients were ambulatory (0% wheelchair, *P* = .003). Before TOFA, 2 patients (20%) were K-level 2 or better, vs all 9 (100%) afterward (*P* < .001). The final mobility comparisons are TUG (Fig. 5) and 6MWT (Fig. 6). Only 2 patients were physically capable of performing preoperative mobility tests; the TUG averaged 14.0 ± 4.5 seconds, and 6MWT averaged 62.6 ± 124 meters. Eight of the 9 living patients completed postoperative mobility tests, averaging a TUG of 17.1 ± 6.9 seconds (*P* = .507) and 6MWT of 232 ± 67 meters (*P* = .004).

**Table 2:** Patient’s mobility assessments.

Pt #	Daily prosthetic wear hours		K-level		Timed Up and Go		6-Min Walk Test	
	Preop	Postop	Preop	Postop	Preop	Postop	Preop	Postop
1	0	11	0	2	WC	10.7	0 (WC)	238
2	Fusion	14	0	3	WC	22.9	0 (WC)	200
3	0	5	0	3	WC	14.2	0 (WC)	263
4	0	8	0	2	WC	9.7	0 (WC)	325
5	N/A	N/A	1	2	N/A	N/A	N/A	N/A
6	0	14	0	3	WC	20.9	0 (WC)	188
7	11	11	2	2	10.8	24.1	263	146
8	Fusion	11	0	2	WC	25.3	0 (WC)	178
9	N/A	16	2	3	17.2	9.2	300	325
10	Fusion	Died	0	Died	WC	Died	0 (WC)	Died
All	≥8 h = 17%	≥8 h = 78%	≥2 = 20%	≥2 = 100%	14.0 ± 4.5	17.1 ± 6.9	62.6 ± 1242	32 ± 67
*P*	***<.041***		***<.001***		.507		***.004***	

N/A, not available; WC, patients confined to their wheelchair.

These patients were assigned a 6MWT value of 0 meters since they could not walk.

Bold values indicate *P* < .05.

**Figure 7:**
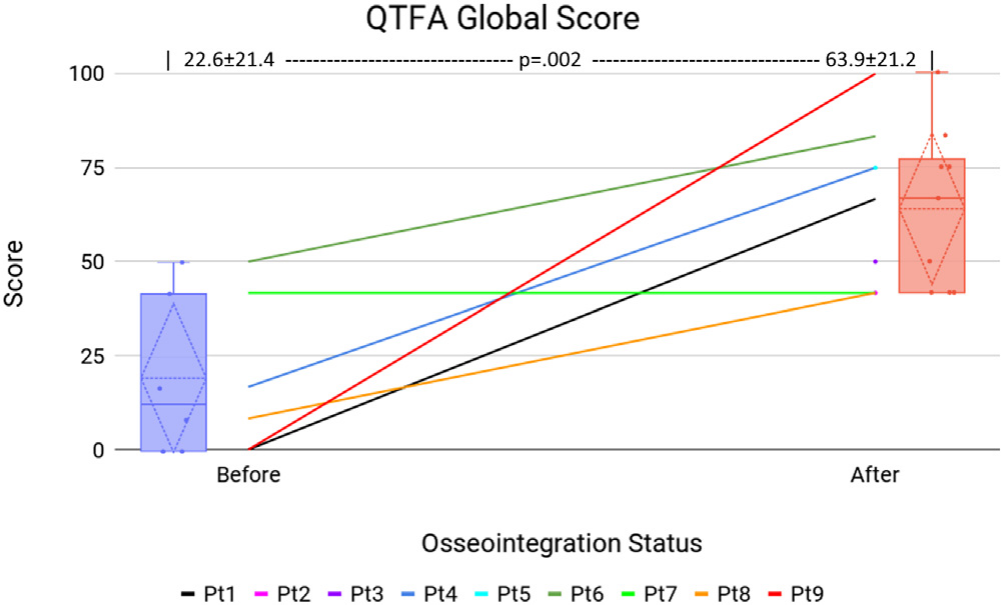
QTFA Global score. The center line graph shows each patient as they were in a socket and at their final evaluation following osseointegration. The color identifying each patient is consistent among all figures in this article. The left and right flanking box plots identify the mean (horizontal dotted line), quartiles (diamond dotted lines), range (whiskers), and each data point (solid dot) of the cohort, representing patient status as they were in a socket and at their final evaluation following osseointegration.

**Figure 8:**
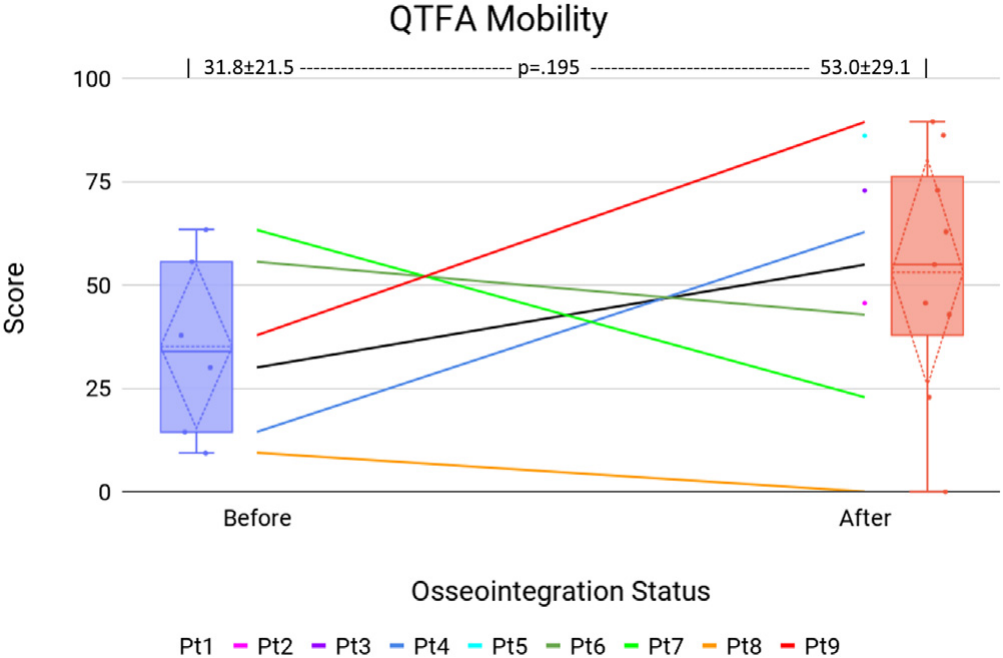
QTFA Mobility score. The center line graph shows each patient as they were in a socket and at their final evaluation following osseointegration. The color identifying each patient is consistent among all figures in this article. The left and right flanking box plots identify the mean (horizontal dotted line), quartiles (diamond dotted lines), range (whiskers), and each data point (solid dot) of the cohort, representing patient status as they were in a socket and at their final evaluation following osseointegration.

**Figure 9:**
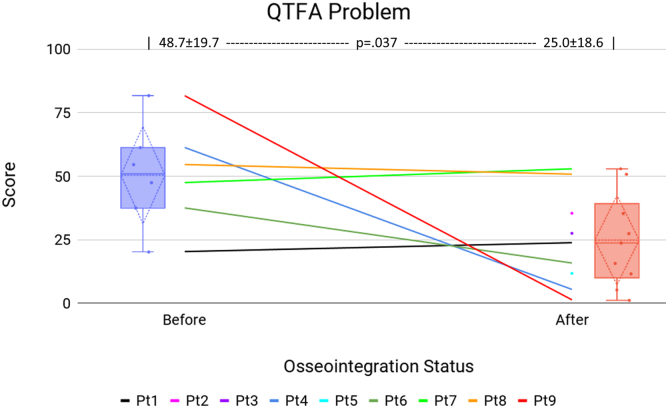
QTFA Problem score (note, lower score is better patient experience). The center line graph shows each patient as they were in a socket and at their final evaluation following osseointegration. The color identifying each patient is consistent among all figures in this article. The left and right flanking box plots identify the mean (horizontal dotted line), quartiles (diamond dotted lines), range (whiskers), and each data point (solid dot) of the cohort, representing patient status as they were in a socket and at their final evaluation following osseointegration.

Table 3 presents patients’ QTFA survey scores for Global (Fig. 7), Mobility (Fig. 8), and Problem (Fig. 9) sections before and after surgery. Significant improvements were observed in the Global score (22.6 ± 21.4 vs 63.9 ± 21.2, *P* = .002) and the Problem score (48.7 ± 19.7 vs 25.0 ± 18.6, *P* = .037). All patients who provided a preoperative Global score improved following TOFA.

**Table 3:** QTFA survey data.

Pt #	Global		Mobility		Problem (lower better)	
	Preop	Postop	Preop	Postop	Preop	Postop
1	0	66.7	30.0	54.9	20.3	23.8
2	Declined	41.7	Declined	45.6	Declined	35.4
3	Declined	50	Declined	72.8	Declined	27.5
4	16.7	75.0	14.4	62.8	61.3	5.4
5	Declined	75.0	Declined	86.1	Declined	11.7
6	50.0	83.3	55.6	42.8	37.5	15.8
7	41.7	41.7	63.3	22.8	47.5	52.9
8^a^	8.3	41.7	9.4	0	54.6	50.8
9	0	100	37.8	89.4	81.7	1.3
10^a^	41.7	Died	12.2	Died	38.3	Died
All	22.6 ± 21.4	63.9 ± 21.2	31.8 ± 21.5	53.0 ± 29.1	48.7 ± 19.7	25.0 ± 18.6
*P*	***.002***		.195		***.037***	

Bold values indicate *P* < .05.

a Although the QTFA is intended for amputees, patients with fused knees completed the survey to assess their mobility and other self-assessment, excluding the specific prosthesis use questions.

### Adverse events

Table 4 presents operative complications after TOFA. Through 1 year, relatively few additional surgeries were required to manage complications. However, after several years, 7 patients (70%) had at least 1 additional surgery, and 5 (50%) had multiple surgeries. Through the final follow-up, there was an average of 1 debridement per patient, and 1.3 refashionings per patient. The single patient whose implant required removal (patient 1) sustained a fall causing a periprosthetic fracture 1 year after the index TOFA, which was managed with open reduction and internal fixation with implant retention [28], and eventually developed the infection over 7 years following that reconstruction. She had a staged explantation with antibiotic spacer placement followed by revision TOFA and has returned to independent ambulation.

**Table 4:** Operative complications after osseointegration.

Pt#	BMI	Follow-up y	Through 1 y			Additional through final follow-up		
			Debridement	Refashioning	Removal	Debridement	Refashioning	Removal
1	39.2	9.7				2@ 4.7 and 5.3 y	3@ 3.0, 4.7, and 5.3 y	1: 8.8 y
2	39.1	5.6		1@ 0.2 y			2@ 1.1 and 5.7 y	
3	37.6	7.4			1: 0.8 y	4@ 1.5, 1.6, 3.5, and 4.0 y	2@ 1.5 and 4.0 y	
4	30.0	6.6	1@ 0.3 y			2@ 2.8 and 5.3 y	3@ 2.7, 4.1, and 5.3 y	
5	36.8	5.1						
6	30.0	5.6				1@ 3.8 y	3@ 1.4, 3.0, and 3.7 y	
7	38.7	5.2				1@ 3.6 y		
8	26.1	3.6	1@ 0.2 y					
9	39.9	2.5						
10	19.9	1						
All	33.7 ± 6.9	5.2 ± 2.5	1 Patient, 1 debridement, 0.3 ± 0.0 y	1 Patient, 1 refashioning, 0.2 ± 0.0 y	1: 0.8 y	5 Patients, 10 debridements, 3.61 ± 1.3 y	5 Patients, 13 refashioning, 3.51 ± 1.5 y	1: 8.8 y

BMI, body mass index.

## Discussion

Overall, this study confirmed the following hypothesis: Patients who have had TFA or KF following infected TKR achieve better mobility and QOL following TOFA. This conclusion is supported by the significant improvements of the pre-TOFA vs post-TOFA mobility (daily prosthesis wear hours, K-level, 6MWT) and QOL surveys (QTFA Global and Problem scores). These improvements are particularly noteworthy given the multiple additional surgeries many patients required, which often reduce a patient’s mobility and reported QOL. It is also remarkable that all 7 patients who were wheelchair-bound at presentation were ambulatory following TOFA (patient 10 was able to walk although he did not complete the formal mobility tests before dying). Because no prior studies present TOFA for patients prior to total joint replacement PJI, instead of evaluating this study’s context among other directly comparable literature, the discussion will focus on how to consider TOFA vs KF or TFA for patients with TKR PJI and summarize important fundamentals of TOFA.

The prognosis of TKR PJI can be grim. The annual mortality risk after TKR PJI managed with 2-stage revision is estimated to be 4.2%, or 21.1% through 5 years [4], worse than the 4 most common cancers: testicular, Hodgkin’s lymphoma, melanoma, and breast cancer [29]. Because KF and TFA with TSP rehabilitation are recognized to provide poor QOL and mobility [1,30], they remain options of last resort, usually following multiple salvage attempts [31]. Unfortunately, delaying and inevitable KF or TFA may worsen patients’ QOL [32]. KF may be viewed as favorable because patients retain the potential for bipedal ambulation without a prosthetic leg. However, QOL may not be superior [33,34]; 5.9% of patients may remain infected, 14.6% may lose ambulatory capacity, 18.8% of ambulatory patients may require assistive devices, and 50% of patients may have additional unplanned surgeries. Regarding TKR PJI amputees, only 25% may walk at 38.5 months following amputation [6], 55.9% primarily use a wheelchair for mobility, 79.5% have phantom pain, 47.1% require chronic pain medications, and only 52.9% were satisfied with their QOL [35]. Indeed, TKR PJI management remains a formidable challenge.

The impaired mobility of KF or TFA substantially contributes to the reduced QOL and survival following TKR PJI. Beyond the gross physical impairment, age is also a factor. Better physical activity and mobility promote mental well-being [36] and reduce vascular risk factors proportional to the metabolic equivalent task [37] in older adults, even at relatively moderate levels such as walking or leisure-time activities [[37], [38], [39], [40]]. The average TKR patient is 65 years old [41], meaning many patients who then develop PJI with subsequent KF or TFA are likely to be even older. A lower extremity amputation, particularly a TFA, requires substantial patient energy expenditure and motivation to remain ambulatory, which is especially demanding for older patients [42]. A review of osseointegration outcomes identified that TOFA is consistently associated with more prosthesis daily wear hours and a lower energy requirement for ambulation [14] and easier short mobility tasks (TUG) and longer distance mobility (6MWT) [43] than TSP rehabilitation. Furthermore, some authors have proposed that for TFA patients older than 60 years, using a prosthesis more than 6 hours daily [44] and walking at least 100 meters [42] represent rather successful rehabilitation. Three of our patients aged 65 years or older had preoperative and postoperative mobility data (patients 4, 6, and 8), following TOFA. They wore their prosthesis 11 ± 3 hours daily and walked 230 ± 82 meters in 6 minutes, which substantially exceeds the proposed benchmark and is particularly notable since all 3 were wheelchair-bound prior to TOFA. From a mobility and QOL standpoint, TOFA appears to be superior to KF and TFA.

Because TOFA patients have an open stoma to connect their prosthetic leg to their residual limb’s bone, they have a potential source of infection that KF and TFA patients do not. Such infections may require oral antibiotics or, in around 7% of patients, operative debridement or implant removal [45,46]. Certainly, these interventions impair mobility and QOL, but no reports of deaths have been reported in association with TOFA infection. It is well documented that the majority of TFA patients using a TSP develop ulcers, dermatitis, or other skin problems that cause periodic prosthesis disuse [47,48]. Literature that identifies rates of operative intervention for such skin issues cannot be found, but anecdotally, operative debridement for TSP-induced skin breakdown is exceptionally rare. Therefore, patients and surgeons must weigh the high likelihood of improved mobility and QOL conferred by TOFA vs the potential of stoma-related infection.

The field of TOFA is relatively young, with the first attempts performed in the 1940s, and the first long-term successful procedure performed in 1990. As such, the basic and clinical sciences are still rapidly progressing. Recent articles [11,49,50] provide in-depth yet clinically oriented explorations of the historical origins, basic science progression, and clinical evolution. Some critical topics are worth highlighting here. The term “osseointegration” can refer to either the phenomenon of an implant remaining clinically solidly fixed in bone over time [51] or also to the surgical procedure of implanting an implant into a patient for reconstruction; that is why this article uses the term TOFA to refer specifically to the procedure for amputees, to differentiate from the biological phenomenon or the procedure for other situations. Titanium alloys (most commonly Ti6Al4V) are currently the material of choice for most commonly used osseointegration implants (particularly in dental procedures but also in bone-anchored hearing aids, in addition to TOFA), but other alloys exist (notably vitallium Co30Cr5Mb). Dental osseointegration was the first clinically successful implementation [52] and remains the most common surgical reconstruction based on this principle. In orthopedic surgery, the most common procedure based on osseointegration principles is uncemented arthroplasty. It is critical to emphasize that although early investigations with light microscopy interpreted that titanium’s exceptional biocompatibility was due to an uninterrupted interface with bone [53], transmission electron microscopy has proven that although collagen fibrils approach the implant surface, a submicron-thick mineral layer permanently remains between the bone and titanium [54,55]. That means titanium (and other osseointegration implants) achieves stability due to exceptionally close bone interdigitation, not true biologic bonds or adhesion. TOFA is a unique medical procedure because it involves a nonbiologic-biologic construct that is transcutaneous, load-bearing, and stable over a long time period and accommodates large motion in all geometric planes. Dental osseointegration is relatively similar, but more consistent and earlier success probably is in part because the teeth do not have the same implant-soft tissue stresses that transcutaneous limb constructs do and likely also due to the unique gingival properties that are more favorable than those of skin. Indeed, the skin seemingly remains the final challenge to providing a total limb replacement [56].

The primary limitation of this study is the small cohort of 10 patients. There was also considerable heterogeneity in the demographic characteristics and prior surgical history among the patients: Some patients had KF, whereas others had TFA, for various durations prior to TOFA. The merits of this study include the minimum 2-year follow-up for all patients (except the deceased patient), along with the attention to mobility and also QOL data metrics. There are several potential sources of bias. TOFA remains relatively obscure and financially expensive, limiting potential patient awareness and access. Furthermore, highly functional and satisfied patients with KF or TFA may not seek TOFA, potentially biasing our selection. No patients were lost to follow-up, so reporting bias is limited. Our team’s high-volume TOFA experience may also bias the reproducibility of the results.

## Conclusion

TOFA may be a preferable rehabilitative alternative for patients with TKR PJI who are facing KF or TFA. Our cohort experienced significantly improved mobility (prosthesis wear hours, K-level, and 6MWT) and QOL (QTFA Global and Problem scores) vs when they had a KF or a TFA with TSP. It is hoped that as adjunctive techniques at the index procedure potentially reduce subsequent surgeries, TOFA can provide an increasingly streamlined preferable alternative to KF or TFA with TSP.

## Conflicts of interest

The authors declare the following financial interests/personal relationships which may be considered as potential competing interests: M. Al Muderis is the sole beneficiary of Osseointegration Holdings Pty Ltd. (OH) and Osseointegration International Pty Ltd. (OI). OI exclusively distributes the OPL implant system worldwide. OH owns the rights and patents to the OPL implant system. The other authors have no declarations.

For full disclosure statements refer to https://doi.org/10.1016/j.artd.2022.04.008.

## Appendix A Supplementary data

Conflict of Interest Statement for Akhtar

Conflict of Interest Statement for Al Muderis

Conflict of Interest Statement for Hoellwarth

Conflict of Interest Statement for Oomatia

Conflict of Interest Statement for Tetsworth
