# Supplementary material for: Osseointegration Following Transfemoral Amputation After Infected Total Knee Replacement: A Case Series of 10 Patients With a Mean Follow-up of 5 Years
Source: Arthroplast Today. 2022 May 21;16:21–30. doi: 10.1016/j.artd.2022.04.008 (PMC9126745; doi:10.1016/j.artd.2022.04.008)
Supplement: Conflict of Interest Statement for Al Muderis [file mmc2.pdf]

INDIVIDUAL CONFLICT OF INTEREST STATEMENT

American Association of Hip and Knee Surgeons

(Adopted from the American Academy of Orthopaedic Surgeons disclosure statement)

The following form **must be filled out completely and submitted by each author (example, 6 authors, 6 forms). All items require a response. If there is no relevant disclosure for a given item, enter "*None*."**

Manuscript Title

1. Royalties from a company or supplier (The following conflicts were disclosed)

Munjed Al Muderis is the sole beneficiary of Osseointegration Holdings Pty Ltd ("OH") and Osseointegration International Pty Ltd ("OI"). OI exclusively distributes the OPL implant system worldwide. OH owns the rights and patents to the OPL implant system

2. Speakers bureau/paid presentations for a company or supplier (The following conflicts were disclosed)

3A. Paid employee for a company or supplier (The following conflicts were disclosed)
None

3B. Paid consultant for a company or supplier (The following conflicts were disclosed)
Munjed Al Muderis is the sole beneficiary of Osseointegration Holdings Pty Ltd ("OH") and Osseointegration International Pty Ltd ("OI"). OI exclusively distributes the OPL implant system worldwide. OH owns the rights and patents to the OPL implant system.

3C. Unpaid consultants for a company or supplier (The following conflicts were disclosed)
None

4. Stock or stock options in a company or supplier (The following conflicts were disclosed)
Munjed Al Muderis is the sole beneficiary of Osseointegration Holdings Pty Ltd ("OH") and Osseointegration International Pty Ltd ("OI"). OI exclusively distributes the OPL implant system worldwide. OH owns the rights and patents to the OPL implant system.

5. Research support from a company or supplier as a Principal Investigator (The following conflicts were disclosed)
None

6. Other financial or material support from a company or supplier (The following conflicts were disclosed)
None

7. Royalties, financial or material support from publishers (The following conflicts were disclosed)
Munjed Al Muderis is the sole beneficiary of Osseointegration Holdings Pty Ltd ("OH") and Osseointegration International Pty Ltd ("OI"). OI exclusively distributes the OPL implant system worldwide. OH owns the rights and patents to the OPL implant system.

8. Medical/Orthopaedic publications editorial/governing board (The following conflicts were disclosed)
None

9. Board member/committee appointments for a society (The following conflicts were disclosed)
None

<u>Each author must sign AND print or type his/her name, date and submit a separate form</u>

In addition, one BLINDED Conflict of Interest form (no author names used) should be submitted per manuscript with all author disclosures.

Munjed Al Muderis		January 9, 2022
Author Name (Print or Type)	Author Signature	Date
